# Effects of GLP-1 Receptor Activation on a Pentylenetetrazole—Kindling Rat Model

**DOI:** 10.3390/brainsci9050108

**Published:** 2019-05-14

**Authors:** Abdelaziz M. Hussein, Mohamed Eldosoky, Mohamed El-Shafey, Mohamed El-Mesery, Khaled M. Abbas, Amr N. Ali, Ghada M. Helal, Osama A. Abulseoud

**Affiliations:** 1 Department of Medical Physiology, Faculty of Medicine, Mansoura University, Mansoura 35516, Egypt; dr_m_dosoky2006@yahoo.com; 2 Department of Human Anatomy, Faculty of Medicine, Mansoura University, Mansoura 35516, Egypt; drmohamedshafey@gmail.com; 3 Department of Anatomy, Fakeeh College of Medical Sciences, Jeddah 2537, Saudi Arabia; 4 Department of Biochemistry, Faculty of Pharmacy, Mansoura University, Mansoura 35516, Egypt; elmesery@hotmail.com; 5 Faculty of Medicine, Mansoura University, Mansoura 35516, Egypt; kmakm_1@outlook.com (K.M.A.); amrnabmans@hotmail.com (A.N.A.); 6 Department of Medical Biochemistry, Faculty of Medicine, Mansoura University, Mansoura 35516, Egypt; ghadahelal76@yahoo.com; 7 Chemistry and Drug Metabolism, IRP, National Institute on Drug Abuse, National Institutes of Health, Biomedical Research Center, Baltimore, MD 21224, USA; osama.abulseoud@nih.gov

**Keywords:** pentylenetetrazole, epilepsy, Hsp70, LC3, caspase-3 and β-catenin

## Abstract

**Objectives**: To study the possible anti-seizure and neuroprotective effect of glucagon like peptide 1 (GLP1) analogue (liraglutide) in a pentylenetetrazole (PTZ) induced kindled rat model and its underlying mechanisms. **Methods**: Thirty Sprague Dawley rats were allocated into 3 equal groups; i) Normal group: normal rats received normal saline, ii) PTZ (kindling) group: received PTZ (50 mg/Kg intraperitoneally (i.p.)) every other day for 2 weeks and iii) PTZ + GLP1 group: same as the PTZ group but rats received liraglutide (75 µg/kg i.p. daily) for 2 weeks before PTZ injection. Seizure severity score, seizure latency and duration were assessed. Also, the expression of caspase-3 (apoptotic marker) and β-catenin (Wnt pathway) by western blotting, markers of oxidative stress (GSH, CAT and MDA) by biochemical assay and the expression of LC3 (marker of autophagy) and heat shock protein 70 (Hsp70) by immunostaining were assessed in hippocampal regions of brain tissues. **Results**: PTZ caused a significant increase in Racine score and seizure duration with a significant decrease in seizure latency. These effects were associated with a significant increase in MDA, β-catenin, caspase-3, Hsp70 and LC3 in brain tissues (*p* < 0.05). Meanwhile, liraglutide treatment caused significant attenuation in PTZ-induced seizures, which were associated with significant improvement in markers of oxidative stress, reduction in LC3, caspase-3 and β-catenin and marked increase in Hsp70 in hippocampal regions (*p* < 0.05). **Conclusion**: Activation of GLP1R might have anticonvulsant and neuroprotective effects against PTZ-induced epilepsy. These effects could be due to suppression of oxidative stress, apoptosis and autophagy and upregulation of Hsp70.

## 1. Introduction

Epilepsy is the most common serious neurological disorder [1] that affects approximately 70 million people of all ages throughout the world [2]. In Egypt, the prevalence of epilepsy was 6.98/1000 [3]. Epilepsy is characterized by persistent spontaneous seizures due to abnormal synchronous neuronal discharges within the brain [4]. Antiepileptic drugs (AEDs) are symptomatic and have more anti-seizure effects than antiepileptic effects [5]. In addition, these drugs have many side effects such as systemic and neurological toxicity, depression, loss of memory and osteoporosis [6]. Therefore, discovery of a novel line of treatment that prevents the epileptic seizure is necessary.

Although several studies have tried to understand the mechanisms underlying the process of epileptogenesis and development of seizures in epilepsy, the pathogenesis of epilepsy still remains poorly understood [7]. Several mechanisms had been postulated to explain the processes underlying pathogenesis of epilepsy including oxidative stress [8,9], apoptosis [8,10,11], gap junction proteins (connexins) [12] and heat shock proteins [13,14]. Autophagy is a regulated process that aims to maintain cell integrity and intracellular homeostasis [15]. Dysfunctions of the process of autophagy have been associated with a variety of diseases such as neurodegenerative disorders [16]. Few studies investigated the potential role of autophagy in epilepsy [17,18]. Wong, [17] reported that the mammalian target of rapamycin (mTOR) pathway of autophagy might be involved in the process of epileptogenesis. Moreover, Wong, [18] demonstrated that genetic inactivation of Atg7, an essential promoter of autophagy, resulted in spontaneous seizures in mice. 

Glucagon-like peptide-1 (GLP-1), primarily secreted from enteroendocrine L cells of GIT, is involved in the regulation of blood glucose [19]. Also, GLP-1 is secreted in small amounts in the hippocampus and caudate nuclei of the brain and its receptors (GLP-1R) are expressed in the neurons [20]. Previous studies demonstrated lowering of the threshold of seizure, reduction of cognitive performance and impairment of synaptic plasticity in GLP-1R deficit mice [21,22]. In addition, Hansen et al., [23] found that liraglutide (GLP1R agonist) increases the memory retention and number of pyramidal neurons in the hippocampus of mice, suggesting its neuroprotective effect. Moreover, while there are several publications that address the beneficial effect of GLP-1 analogues in epilepsy, to the best of our knowledge none of them discuss the relation between GLP-1 analogues and autophagy in epilepsy. To the best of our knowledge there are still only two reports by Koshal and Kumar, [24,25] about the use of liraglutide in protecting against epilepsy, but none of them discuss the autophagy, Hsp70 and β-catenin as a molecular mechanism for GLP1 in ameliorating epilepsy. Therefore, we investigated in the present study the possible anti-epileptic effect of liraglutide (GLP-1R agonist) against PTZ kindled epilepsy and its possible underlying mechanisms by targeting autophagy, apoptosis, oxidative stress and the Wnt pathway.

## 2. Materials and Methods

### 2.1. Animals

Thirty male Sprague Dawley rats weighing 190 ± 10 g and aged 5–6 months were included in the present study. Rats were individually housed in separate cages at the Physiology Department, Mansoura College of Medicine. Animals were fed on standard chow and had free access to water. All experimental procedures and protocols were approved by and done according to the guidelines of the Institutional Beview board (IRB) of the Mansoura College of Medicine (# r/17.01.102).

### 2.2. Study Design

Rats were randomly allocated into 3 groups (each 10 rats) as follows; i) Normal group: were normal rats that received saline (0.2 mL via i.p. injection), ii) PTZ group: rats received PTZ (50 mg/kg i.p in 0.2 mL saline) every other day for 2 weeks and iii) GLP1 + PTZ group: as PTZ group but rats received liraglutide 75 µg/kg daily via i.p. and right before PTZ injection at the days of PTZ injection for 2 weeks i.e., 7 injections of PTZ and 14 injections of GLP1. 

### 2.3. PTZ-Induced Kindled Rat Model and Behavioral Assessment

Kindling of rats with PTZ was done by i.p. injection of rats with PTZ (50 mg/kg) every other day for 2 weeks. Full rat kindling was achieved when the rats had stage 4 or 5 for 3 successive doses of PTZ [14]. After PTZ injection, the behavioral changes of rats were video recorded for 30 min. The records were analyzed blindly for the seizure’s stage, latency of the seizure onset (sec) and duration of seizure (sec). The severity of seizures was scored according to a well-established Racine’s scoring scale [26]. 

### 2.4. Harvesting of Brain Specimens

By the end of the experiment, rats were euthanatized by overdose of Na^+^ thiopental (120 mg/kg i.p.). Transcardiac catheter was used to perfuse the brain by 100 mL heparinized saline followed by 150 mL of 10% formalin to remove blood clots and to fix the brain specimens. Then, the harvested brain tissues were fixed in formalin (10%) for 4 h and stored in a sucrose (25%) solution containing 0.1% sodium azide until the time of histopathological study and immunostaining. Also, brain specimens for biochemical analysis for oxidative stress markers and western blotting were harvested after saline perfusion only and were stored in liquid nitrogen until the time of analysis.

### 2.5. Measurement of Oxidative Stress Markers (MDA, GSH and Catalase Activity) in Brain Tissues

By using mortar and pestle, about 50–100 mg of brain tissues were homogenized in 1–2 mL cold buffer (50 mM potassium phosphate, pH 7.5, 1 mM EDTA) and centrifuged for 15 min at 4000 rpm (4 °C). The concentrations of MDA and GSH and the activity of catalase enzyme were measured in the supernatant of brain homogenates using commercially available kits (Bio-Diagnostics, Giza, Egypt) according to the manufacturer’s instructions. 

### 2.6. Measurement of β-Catenin and Caspase-3 Protein Expression by Western Blotting

Formation of total cell lysate from brain tissues, measurement of protein concentrations in samples, vertical SDS-PAGE gel electrophoresis and western blotting as well as the cat# of primary and secondary antibodies of β-catenin, caspase-3 and β-tubulin were discussed in detail in our previous work [27]. 

### 2.7. Histopathological Examination for Hippocampal Neurons in CA3 Region

After fixation of brain tissues in paraffin block, 20 µm thick slides were processed and stained with hematoxylin and eosin (H & E). After staining, the CA3 region of hippocampus was examined under light microscope (Leica DM500 with camera Leica ICC50HD, Leica Microsystems, Tokyo, Japan) blindly for loss of neurons and pyknotic nuclei. 

### 2.8. Measurement of Hsp70 and LC3 Expression by Immunohistochemistry

Forty micrometer serial sections of brain specimens were obtained using a freezing sledge microtome for immunohistochemical examination. All steps of peroxidase-based immunostaining staining were mentioned in detail in our previous work [27]. The primary antibodies include polyclonal anti-LC3 rabbit antibody (Cat#YPA1340, dilution 1:200; Chongqing Biospes Co, Ltd., Chongqing, China) and polyclonal rabbit anti-heat shock protein 70 (Zymed). Pictures were captured using an Olympus^®^ digital camera connected to an Olympus^®^ microscope with a 1/2 × photo adaptor, using a 40 × objective (Leica Microsystems, Tokyo, Japan). We prepared five slides from each case and five random fields were examined in each slide. The density of the positively stained area was estimated.

### 2.9. Statistical Analysis

Statistical analysis was done using GraphPad Prism version 7 software, (La Jolla, CA, USA). Data of behavioral parameters and molecular parameters are presented as mean ± standard errors of mean (SEM). Repeated measures analyses of variance (ANOVA) with Tukey posthoc *t*-tests were used to study the effect of treatment and time factors on behavioral variables. Two separate survival analyses were used to examine differences in latency to first jerk and seizure duration between the two groups. One-way analyses of variance (ANOVA) with a Scheffe posthoc test was used to find statistically significant differences among the three studied groups. *p* value ≤ 0.05 is considered statistically significant. 

## 3. Results

### 3.1. Animal Survival

Whilst no deaths were recorded in the normal group, one rat in the PTZ group and two rats in GLP1 + PTZ groups died.

### 3.2. Neurobehavioral Changes

**Figure 1:**
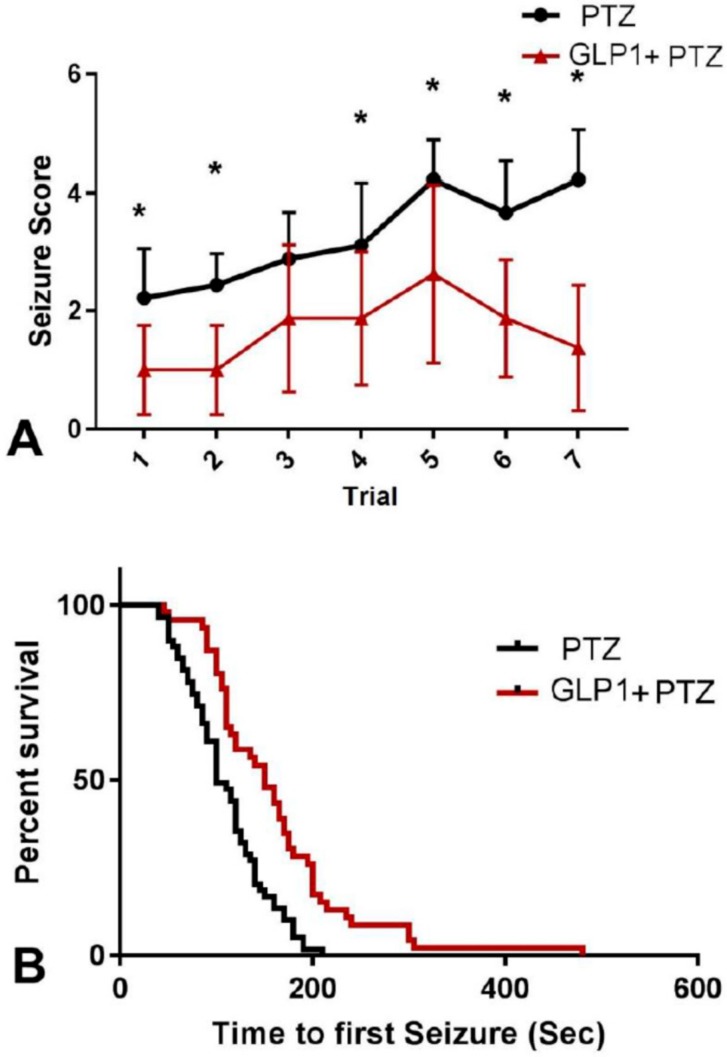

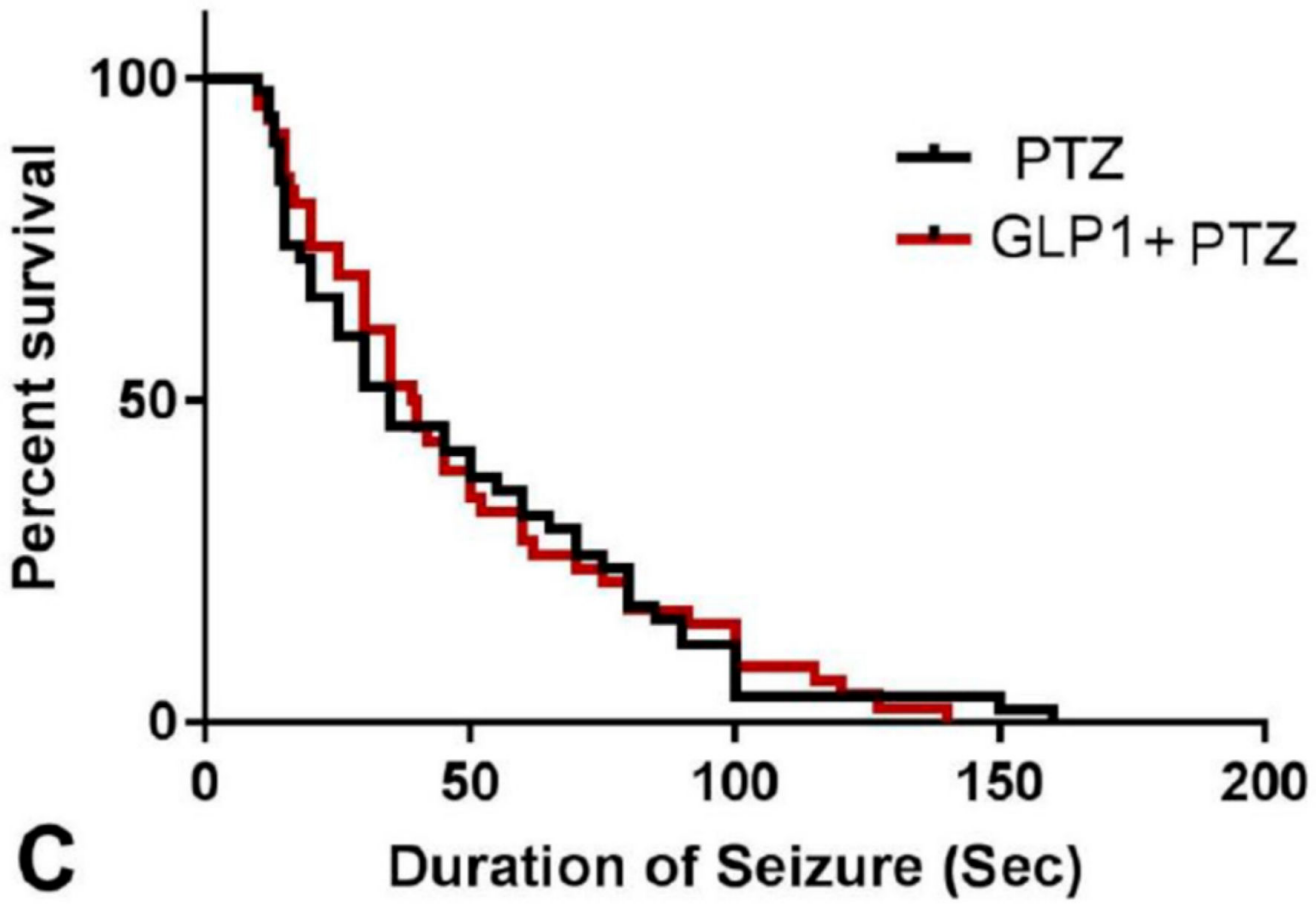
The behavioral effects of GLP1 on PTZ-induced seizures. (**A**) = Seizure score, and Survival analysis vs time to first seizure (**B**) and seizure duration (**C**). Two-way ANOVA test. *, Significant vs PTZ + Sal.

Administration of GLP1 in PTZ-treated rats caused marked reduction in seizure score (*F* (1,15) = 128.6, *p* < 0.0001) that was evident in early treatment (GLP1 + PTZ group vs. PTZ group trial 1 mean ± SEM = 1.0 ± 0.2 vs. 2.2 ± 0.2, *t* = 3.15 *df* = 15, *p* = 0.006) and continued throughout treatment (trial 7: 1.3 ± 0.3 vs. 4.2 ± 0.2, *t* = 6.19 *df* = 15, *p* < 0.0001, Figure 1A). Also, GLP1 treatment significantly prolonged the latency to first seizure (median survival time GLP1 + PTZ group vs. PTZ group = 150 vs. 100 s, χ^2^ = 17.84, *df* = 1, *p* < 0.0001, Figure 1B) but did not have an effect on the seizure duration (median survival time GLP1 + PTZ group vs. PTZ group = 39.5 vs. 35 s, χ^2^ = 0.004, *df* = 1, *p* = 0.9, Figure 1C).

### 3.3. Markers of oxidative stress (MDA) and antioxidants (CAT and GSH)

**Figure 2:**
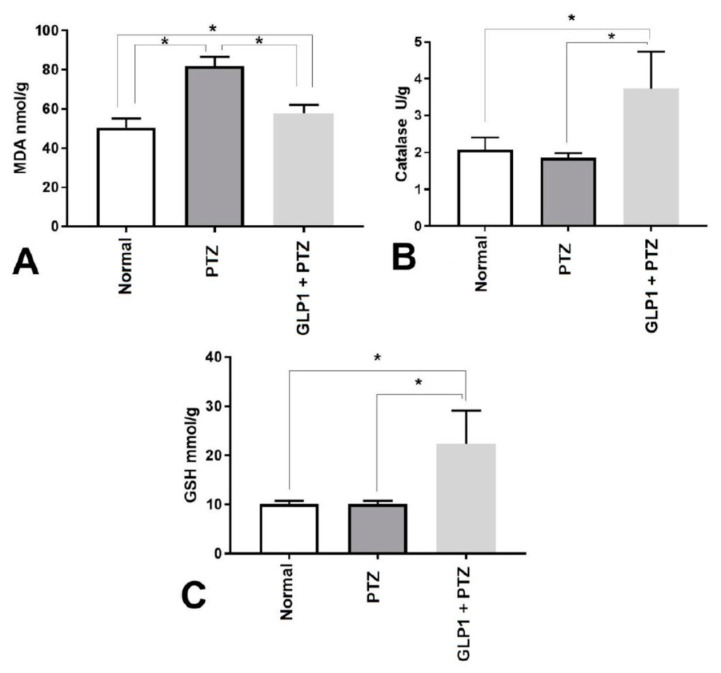
Effects of GLP1 on lipid peroxidation product (MDA concentration (nmol/g brain tissue) (**A**), and catalase enzyme activity (U/g brain tissues) (**B**) and reduced glutathione (GSH) (mmol/g brain tissues) (**C**). One-way ANOVA with Tukey posthoc test. *, Statistical significant difference between two groups.

GLP1 treatment for 14 days attenuated PTZ-induced increase in MDA concentrations (*F* (2,15) = 77.28, *p* < 0.0001, Figure 2A) and increased the activity of catalase enzyme (*F* (2,15) = 16.5, *p* = 0.0002, Figure 2B) and increased GSH concentrations (*F* (2,15) = 19.34, *p* < 0.0001, Figure 2C) in rat brain tissues. 

### 3.4. Expression of Caspase-3 and β-Catenin Proteins by Western Blotting

**Figure 3:**
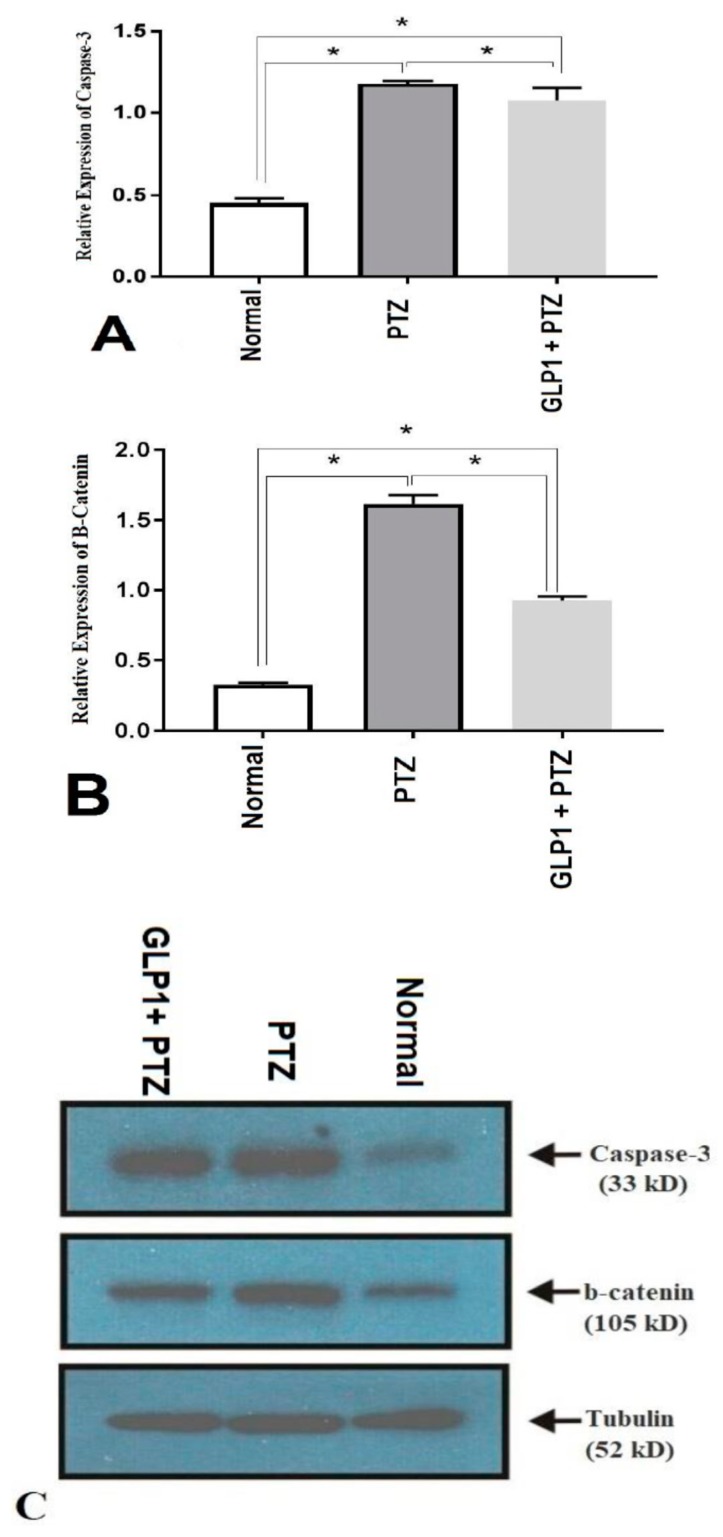
Effects of GLP1 on apoptotic marker (caspase-3) and β-catenin. Score of expression of caspase-3 (**A**), β-catenin (**B**), and **(C)** products of western blotting of caspase-3 and β-catenin and control gene protein (tubulin) in different studied groups. One-way ANOVA with Tukey posthoc test. *, Statistically significant difference between two groups.

PTZ-induced elevation in caspase-3 protein expression was significantly reduced in the group treated with GLP1 (*F* (2,15) = 396.4, *p* = 0.005, Figure 3A). Also, β-catenin was significantly reduced in the GLP1 treated group (*F* (2,15) = 1607, *p* < 0.0001, Figure 3B). Figure 3C shows the bands of western blotting products of caspase-3, β-catenin and tubulin proteins from different groups and their MW in kilo Dalton.

### 3.5. Histopathological Examination of CA3 Region of Hippocampus

**Figure 4:**
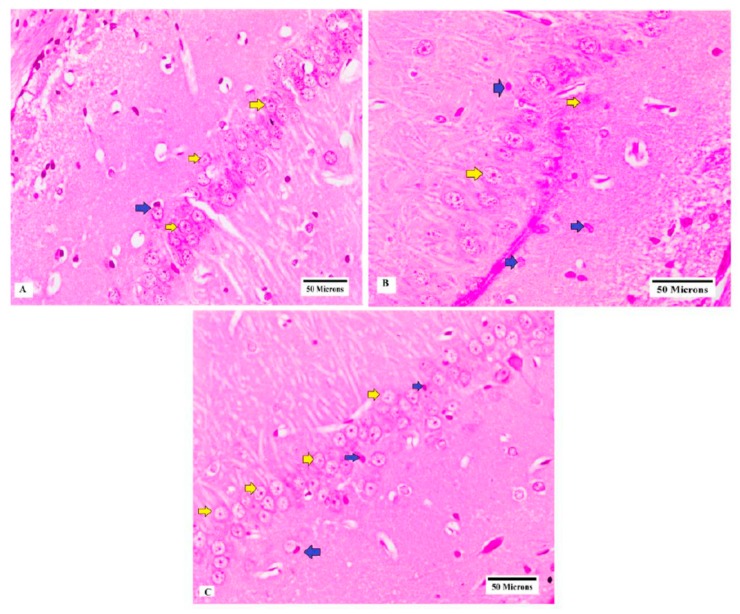
Brain specimens of CA3 hippocampal region showing (**A**) large number of neurons with normal shapes (yellow arrows) (normal group), (**B**) low number of neurons with normal shapes (yellow arrows) and large number of neurons with pyknotic changes and dead neurons (blue arrows) (PTZ group) and (**C**) large number of normal neurons (yellow arrows) with minimal pyknotic changes (blue arrows) (GLP1 + PTZ group) (H & E, 400×).

Brain slides from rats of normal group showed normal number and shape of neurons in CA3 region of hippocampus (Figure 4A), while brain specimens obtained from PTZ group showed neuronal loss and irregularly arranged neurons and pyknotic changes in neurons (Figure 4B). Moreover, brain specimens obtained from the GLP1 + PTZ group showed a large number of neurons with normal shapes (Figure 4C).

### 3.6. Expression of Hsp70 and LC3 by Immunohistochemistry

**Figure 5:**
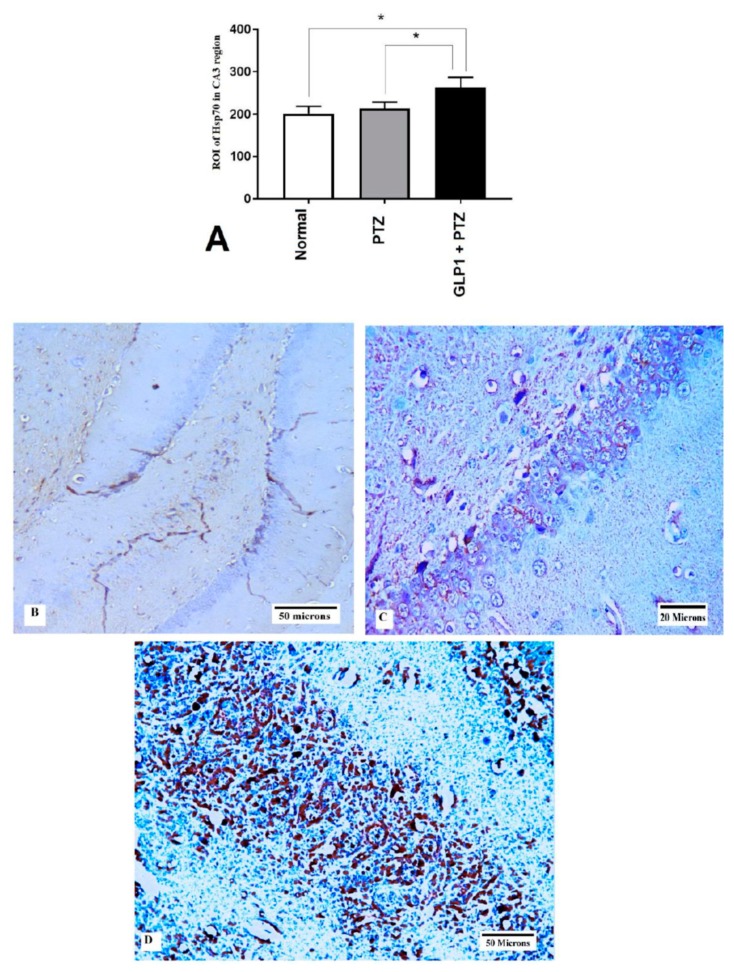
Effects of GLP1 on Hsp70 expression in CA3 hippocampal region. Score of Hsp70 expression in CA3 hippocampal region (**A**), brain sections shows negative expression of HSP70 in CA3 hippocampal region in normal group (**B**), brain sections showing mild HSP70 expression in CA3 hippocampal region in PTZ group (**C**) and brain sections showing marked HSP70 expression in CA3 hippocampal region in GLP1 + PTZ group (**D**). One-way ANOVA with Tukey posthoc test. *, statistically significant difference between two groups.

**Figure 6:**
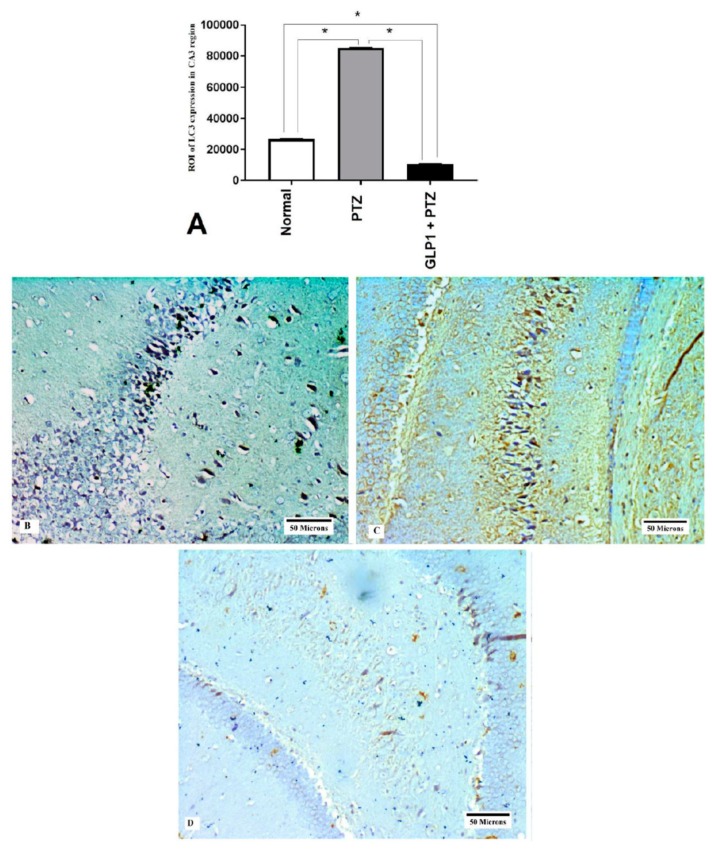
Effects of GLP1 on autophagic marker (LC3) expression in CA3 hippocampal region. Score of expression of LC3 in different groups (**A**), brain sections shows negative expression of LC3 in CA3 hippocampal region in normal group (**B**), brain sections showing marked LC3 expression in CA3 hippocampal region in PTZ group (**C**) and brain sections showing mild LC3 expression in CA3 hippocampal region in GLP1 + PTZ group (**D**). One-way ANOVA with Tukey posthoc test. *, Statistically significant difference between two groups.

GLP1 administration significantly increased the expression of Hsp70 (*F* (2,15) = 17.89, *p* = 0.0001, Figure 5A). Figure 5B–D shows the expression of Hsp70 in CA3 hippocampal region from different groups. Also, GLP1 administration significantly attenuated the PTZ-induced increase in LC3 (*F* (2,15) = 144106, *p* < 0.0001, Figure 6A). Figure 6B–D shows the expression of LC3 in CA3 hippocampal region from different groups.

### 3.7. Correlations

The seizure stage showed significant positive correlation with MDA, Caspase-3, LC3 and beta catenin levels (*p* ≤ 0.031) and negative correlation with GSH and Hsp70 (*p* ≤ 0.041). Also, MDA showed significant correlation with caspase-3 and LC3 (*p* ≤ 0.01) and negative correlation with GSH, Hsp70 and beta catenin (*p* ≤ 0.012). Moreover, LC3 showed significant positive correlation with beta catenin and caspase-3 (*p* ≤ 0.032) with negative correlation with GSH and catalase (*p* ≤ 0.008) (Table 1)

## 4. Discussion

The main findings of the present study can be summarized as follows: i) administration of PTZ at a dose of 50 mg/kg every other day for 2 weeks caused rat kindling which was associated with enhanced oxidative stress, upregulation of LC3, caspase-3, β-catenin and Hsp70 in brain tissues, and ii) treatment with liraglutide with PTZ caused significant attenuation of PTZ-induced seizures and reduction of oxidative stress and downregulation of LC3, caspase-3, β-catenin and Hsp70.

The present study demonstrated a significant increase in seizure stage and duration with shortening of the latency to the first jerk. In our search for alternative epilepsy treatments, liraglutide which is a newer long lasting GLP1 receptor agonist, is an intriguing candidate. It has been demonstrated that systemic administration of liraglutide can penetrate the blood brain barrier (BBB) in experimental animals [28]. Also, liraglutide exerts a neuroprotective effect in several animal models such as Parkinson’s, Alzheimer’s and Huntington’s disease [29]. We found in the present study that repeated daily administration of liraglutide (75 µg/kg) reduced the seizure score and duration and prolonged the latency of seizure. In agreement with these findings, recent studies revealed that liraglutide (75 and 150 μg/kg) was able to inhibit the seizure severity and restored behavioral activity in PTZ and corneal—kindled mice models [24,25]. 

The role of oxidative stress in development and progression of epileptic seizure has been proposed [8,14]. The brain is characterized by an elevated oxidative metabolism and low antioxidants defense mechanisms. Although these antioxidant mechanisms may be sufficient under normal conditions, the brain is vulnerable to oxidative stress during seizures. Moreover, the hippocampus has a low level of vitamin E that is considered a vital anti-oxidant and therefore, it is highly sensitive to oxidative stress [30]. In line with previous studies [14,31,32], we found that PTZ kindling caused a significant increase in redox state in brain tissues, as evidenced by significant increase in MDA concentration that is a product of lipid peroxidation and indicates the level of oxidative stress. These findings suggest the important role of ROS in the pathophysiology of PTZ-induced epilepsy. Also, we found that liraglutide treatment caused significant attenuation of redox state in PTZ-kindled rats. This is evidenced by significant reduction in MDA with significant increase in CAT activity and GSH concentration in brain tissues. In agreement with these findings; two studies by Koshal and Kumar [24,25] demonstrated that liraglutide prevented the formation of ROS and reactive nitrogen species in corneal kindled and PTZ kindling mice. Thus, conclusively, the liraglutide antiepileptic effect may depend partly on its antioxidant activity.

Autophagy is a tightly regulated process in which the intracellular protein aggregates and damaged organelles are degraded and removed by the lysosomal pathway [33]. The present study examined the expression of LC3 proteins in CA3 region of hippocampus, which is considered the most reliable cellular marker for autophagy activation [34]. We demonstrated little expression of LC3 in CA3 region in brains from normal rats and that its expression became highly increased in brains obtained from PTZ group. Moreover, there was a positive correlation between stage of seizure and LC3 expression, suggesting its role in PTZ-induced epilepsy model of chronic epilepsy. Previous studies reported similar findings in other experimental models of epilepsy [35,36]. Shacka et al., [35] found that induction of repeated seizures by kainate caused significant increase in LC3 positive autophagy vacuoles in the hippocampus of mice [35]. Also, Cao et al., [36] found an induction of autophagy in pilocarpine-induced status epilepticus (SE) models. Although, some authors discussed the role of mTOR and Ag7 in epileptogenesis, in the present study we did not investigate the effect of GLP1 on these pathways, which is considered one of the limitations of this study. 

In the present work, we hypothesized that induction of autophagy might be due to ROS production and oxidative stress in brain tissues due to a positive correlation between LC3 and MDA levels in brain tissues. In consistence with this hypothesis, some investigators reported that ROS are important activators of autophagy [37,38]. The upregulation of autophagy (increased expression of LC3 proteins) in the present work could be in response to various neurological insults and is likely a component of excitotoxicity, which contributes to cell death [39,40,41]. Koike et al., [42] concluded that inhibition of autophagy prevents neuronal cell death in neonatal hypoxic-ischemic injury in mice. According to this speculation; inhibition of autophagy by GLP1 is beneficial which is demonstrated in the present study. This is in line with Dong et al., [30] who revealed that oxidative stress resulted in cell loss and induced autophagy, leading to aggravated cell death in hippocampus that was recovered by a natural antioxidant arachidonic acid (AA).

In addition, Cao et al. [43] identified for the first time a role of chaperone-mediated autophagy (CMA) as a part of the seizure stress response. Moreover, inhibition of CMA using antioxidants such as vitamin E may represent a therapeutic target. On the other hand, autophagy is triggered during the PTZ-induced kindling process to get rid of unwanted protein and damaged organelles produced by oxidative stress as an attempt to restore relaxed neurons and suppress the kindling development. This scenario has been proposed by many investigators such as Hosseinzadeh et al. [44] who found that seizures could trigger pathological alterations associated with autophagy impairment and that the use of components activating autophagy might be useful. Consistently, Fornai et al. [45] and Caldero’ et al. [46] mentioned that neuronal damage induced by kainate can be worsened by autophagy blockers while it is prevented by autophagy inducers. Also, Wang et al., [7] demonstrated that curcumin protects neurons in post-SE rat hippocampus through induction of autophagy and inhibition of necroptosis. On the other hand, while some authors showed reduction, other authors showed upregulation in autophagy with GLP-1 treatment [47]. In animal model of hepatic steatosis, Zhou et al. [48] demonstrated that liraglutide caused significant increase in autophagy, which reduces the lipid accumulation in liver; while Chen et al. [49] revealed the protective role of autophagy induced by liraglutide in preventing high glucose level induced insulinoma (INS-1) cell apoptosis. On the other hand, Zhao et al., [33] revealed that liraglutide had a renoprotective effect that could be attributed to inhibition of autophagy. 

Regarding apoptosis, the present study demonstrated induction of neuronal apoptosis in epilepsy, as evidenced by increased expression of caspase-3 in CA3 region of hippocampus of PTZ group. In addition, treatment with liraglutide caused significant reduction in the expression of caspase-3 in the hippocampal regions. These findings are in agreement with previous studies that demonstrated induction of apoptosis in hippocampal regions CA1 and CA3 in KA-induced epilepsy [8,10]. Likewise, expression caspase-3 and induction of neuronal apoptosis were observed in adult rats induced epileptic seizures with PTZ [11]. The oxidative stress caused by excessive production of ROS and disruption of mitochondrial membrane potential might activate the mitochondrial pathway for apoptosis [50]. While the present study revealed upregulation of caspase-3 in PTZ—treated brain, activation of GLP1R failed to significantly attenuate the upregulated caspase-3. Downregulation of caspase-3 by liraglutide might be due to the ability of GLP1 analogues to induce antiapoptotic proteins such as B-cell lymphoma 2 (Bcl-2) [51,52].

Heat shock proteins (Hsps) are molecular chaperones that play an important role in cellular responses to stress [53] and their expression has been detected in many cell types in the nervous system such as neurons, glial and endothelial cells [54]. Several previous studies investigated the expression of Hsp70 in epilepsy and found a direct relationship between seizure frequency, duration, intensity, and Hsp70 expression has been explored in animal models [14,55] and human epilepsy [13,56], suggesting its role as endogenous protective molecule for brain cells during epilepsy. The current study revealed a non-significant increase in the expression of Hsp70 in CA3 region of the hippocampus and a negative correlation with the seizure stage. In addition, this study found a significant increase in Hsp70 expression in CA3 region of hippocampus after GLP1-treatment, suggesting that upregulation of Hsp70 might be a potential mechanism for the neuroprotective effect of GLP-1 against epileptic seizures. Li et al., [57] and Zhao et al., [58] reported that induction of Hsp70 has neuroprotective role in preventing apoptosis. The inducible Hsp70 could prevent the apoptotic processes by interacting with p53, which stimulates the apoptotic cell death [59,60]. Also, Kanitkara and Bhonde [61] demonstrated that Hsp70 can attenuate the oxidative stress in pancreatic beta cells and increase the glucose-induced insulin release. 

The Wnt-β-catenin signaling pathway plays a crucial role in regulation of the fetal development, the process of neurogenesis, neural differentiation, synapse development, and plasticity in the central nervous system (CNS) [62]. Wnt signaling is involved in several CNS disorders such as Alzheimer’s disease, schizophrenia, and mood disorders [63,64,65]. Previous studies reported controversies results regarding the role of the Wnt-β-catenin pathway in epilepsy. In addition, some studies reported upregulation in β-catenin in epileptic animal models [27,66] and the electroconvulsive seizure rat model [67], while other studies found downregulation of β-catenin during epileptic animal models such as Busceti et al. [68] and Goodenough et al., [69] in Kainite-induced rat. In agreement with the first group, we found upregulation of β-catenin in hippocampal regions of the PTZ group that was proposed to be due to oxidative stress. Downregulation of β-catenin in the GLP-1 group compared to the PTZ group supported this hypothesis. Also, in agreement with our results, Campos et al. [70] highlighted the important role of β-catenin in controlling seizure susceptibility to PTZ by using β-catenin knockout mice.

## 5. Conclusions

The current study suggests anti-seizure action for GLP1 against PTZ-induced seizures, which might be due to upregulation of Hsp70, downregulation of autophagy, apoptosis and β-catenin. Further studies are necessary to fully understand the role of autophagy in epilepsy.

## Figures and Tables

**Table 1:** Correlation between seizure stage and MDA, GSH, CAT, caspase-3, β-catenin, LC3 and Hsp70 in PTZ group.

		Stage of Seizure	MDA	GSH	CAT	Caspase-3	β-Catenin	Hsp70	LC3
Seizure stage	*r*		0.77	−0.73	−0.35	0.64	0.64	−0.62	0.66
	*p*		0.001	0.021	0.090	0.004	0.031	0.041	0.012
MDA	*r*			−0.810	−0.45	0.87	0.65	−0.73	0.74
	*p*			0.010	0.066	0.004	0.012	0.009	0.01
GSH	*r*				0.31	−0.81	0.84	0.66	−0.72
	*p*				0.22	0.006	0.002	0.003	0.004
CAT	*r*					−0.14	−0.31	0.62	−0.66
	*p*					0.61	0.22	0.01	0.008
Caspase-3	*r*						0.67	−0.61	0.67
	*p*						0.009	0.041	0.032
β-catenin	*r*							0.61	0.60
	*p*							0.009	0.009
Hsp70	*r*								−0.33
	*p*								0.076

MDA = malondialdehyde, GSH = reduced glutathione concentration, CAT = catalase activity, Hsp70 = heat shock protein 70; *r*, Pearson’s correlation coefficient, *p* < 0.05 is considered significant.
